# Mapping of exogenous choline uptake and metabolism in rat glioblastoma using deuterium metabolic imaging (DMI)

**DOI:** 10.3389/fncel.2023.1130816

**Published:** 2023-04-28

**Authors:** Kevan L. Ip, Monique A. Thomas, Kevin L. Behar, Robin A. de Graaf, Henk M. De Feyter

**Affiliations:** ^1^Department of Radiology and Biomedical Imaging, Magnetic Resonance Research Center, Yale University, New Haven, CT, United States; ^2^Department of Psychiatry, Magnetic Resonance Research Center, Yale University, New Haven, CT, United States; ^3^Department of Biomedical Engineering, Yale University, New Haven, CT, United States

**Keywords:** metabolic imaging, deuterium, choline, glioblastoma, cancer

## Abstract

**Introduction:**

There is a lack of robust metabolic imaging techniques that can be routinely applied to characterize lesions in patients with brain tumors. Here we explore in an animal model of glioblastoma the feasibility to detect uptake and metabolism of deuterated choline and describe the tumor-to-brain image contrast.

**Methods:**

RG2 cells were incubated with choline and the level of intracellular choline and its metabolites measured in cell extracts using high resolution ^1^H NMR. In rats with orthotopically implanted RG2 tumors deuterium metabolic imaging (DMI) was applied *in vivo* during, as well as 1 day after, intravenous infusion of ^2^H_9_-choline. In parallel experiments, RG2-bearing rats were infused with [1,1′,2,2′-^2^H_4_]-choline and tissue metabolite extracts analyzed with high resolution ^2^H NMR to identify molecule-specific ^2^H-labeling in choline and its metabolites.

**Results:**

*In vitro* experiments indicated high uptake and fast phosphorylation of exogenous choline in RG2 cells. *In vivo* DMI studies revealed a high signal from the ^2^H-labeled pool of choline + metabolites (total choline, ^2^H-tCho) in the tumor lesion but not in normal brain. Quantitative DMI-based metabolic maps of ^2^H-tCho showed high tumor-to-brain image contrast in maps acquired both during, and 24 h after deuterated choline infusion. High resolution ^2^H NMR revealed that DMI data acquired during ^2^H-choline infusion consists of free choline and phosphocholine, while the data acquired 24 h later represent phosphocholine and glycerophosphocholine.

**Discussion:**

Uptake and metabolism of exogenous choline was high in RG2 tumors compared to normal brain, resulting in high tumor-to-brain image contrast on DMI-based metabolic maps. By varying the timing of DMI data acquisition relative to the start of the deuterated choline infusion, the metabolic maps can be weighted toward detection of choline uptake or choline metabolism. These proof-of-principle experiments highlight the potential of using deuterated choline combined with DMI to metabolically characterize brain tumors.

## 1. Introduction

Choline (Cho) is a nutrient similar to B vitamins that is almost exclusively obtained through diet. It is an essential component in the synthesis of phospholipids which are key building blocks of cell membranes. In proliferating tissue, such as growing tumors, demand for Cho is often increased, resulting in upregulated transport and metabolism (Glunde et al., 2011). Due to its important role in tumor growth, inhibiting Cho metabolism has been studied as a possible therapeutic strategy, while Cho transport has been a target for tumor imaging (Mertens et al., 2010; Arlauckas et al., 2016; Schiaffino-Ortega et al., 2016; Trousil et al., 2016; Sola-Leyva et al., 2019). The detection of ^11^C-labeled Cho is FDA-approved for positron emission tomography (PET) imaging in suspected recurrent prostate cancer (Medicine S of N, 2012), and this agent and ^18^F-labeled Cho variants are being studied for PET imaging in other cancers as well (Talbot et al., 2014; Bolcaen et al., 2015; Radzikowski et al., 2022). While there are concerns of limited tissue-specificity for this application, PET detection of radio-labeled Cho for characterization of tumor lesions in the brain is an area of active research (Calabria et al., 2018; Lovinfosse et al., 2020).

The detection of increased endogenous Cho levels in brain tumors using ^1^H magnetic resonance spectroscopy (MRS) is one of the earliest described applications of metabolic imaging of brain tumor lesions, introduced more than 30 years ago (Bruhn et al., 1989). The spectral overlap of the peaks of free Cho and its metabolites phosphocholine (PC) and glycerophosphocholine (GPC) prevent their individual detection. However, research studies have repeatedly demonstrated the potential benefits of detecting the overlapping peaks of the combined, total pool of Cho and its metabolites (tCho) as an imaging biomarker of brain tumor grade and treatment response, and marker of malignancy in breast cancer (Katz-Brull et al., 2002a; Oh et al., 2004; Li et al., 2013; Rapalino and Ratai, 2016). Increased tCho levels detected with ^1^H MRS are believed to at least in part reflect an increased membrane turnover in cancer (Glunde et al., 2004). Technical developments have led to the widespread availability on clinical MR imaging (MRI) scanners of methods for single voxel ^1^H MRS and MR spectroscopic imaging (MRSI), which allows spatial mapping of metabolites. Despite the technical improvements and increased availability of ^1^H MRSI methods on clinical MRI scanners, these methods are time-consuming and not very robust, aspects that contribute to the limited use of ^1^H MRSI in the clinic. Furthermore, the increase of tCho in brain tumor tissue relative to normal brain is often relatively small, which results in limited tumor-to-brain image contrast. The limited image contrast is mitigated by displaying tCho as ratio with the level of N-acetyl aspartate (NAA). NAA is a neuronal marker that noticeably decreases when neurons are damaged or replaced by other cell types, but the loss of NAA is common in many neurological diseases and not unique to the presence of tumor cells. The image contrast of tCho/NAA is therefore mostly driven by the lower levels of NAA, and while normalizing tCho with NAA increases the tumor-to-brain image contrast, it comes at the cost of reduced specificity.

As an alternative to ^1^H MRSI-based detection of endogenous tCho levels, we explore a novel approach to detect uptake and metabolism of exogenous, stable isotope-labeled Cho by brain tumors, using the recently described technique of deuterium metabolic imaging (DMI) (De Feyter et al., 2018). DMI is a ^2^H MRSI-based technique that is combined with administration of a deuterated substrate. The method is very robust compared to ^1^H MRSI methods because it does not require water or lipid suppression, or advanced localization schemes. Here we used DMI combined with the intravenous infusion of Cho that is deuterated on its nine equivalent trimethyl proton positions. We first explore Cho uptake in cancer cells *in vitro* before testing the image contrast between tumor and normal brain in a rat model of glioblastoma (GBM). We further characterize the image contrast observed 24 h after initial ^2^H_9_-Cho infusion and show how the timing of DMI data acquisition can result in metabolic maps that are weighted toward transport or metabolism of Cho.

## 2. Materials and methods

### 2.1. *In vitro*

Rat glioma cells, RG2 (American Type Culture Collection, Manassas, VA, USA) and mouse glioma cells, Gl261 (National Cancer Institute, NCI, Bethesda, USA) were grown in T75 flasks using Dulbecco’s modified Eagle’s medium (DMEM) and standard cell culture conditions (37 °C, 5% CO_2_). For *in vitro* Cho uptake experiments cells were incubated with medium containing 1 mM unlabeled choline chloride (MilliporeSigma, Burlington, MA, USA). Cells were harvested at 0, 10, 60, and 120 min of incubation, suspended in saline, pelleted by centrifugation, boiled and metabolites extracted from the supernatant following cell lysis. The supernatant was dried under nitrogen flow (Turbovap, Biotage, Salem, NH, USA). Samples were resuspended in phosphate-buffered (pH 7) NMR buffer containing standards for chemical shift (formate) and concentration (imidazole), and 10% D2O.

### 2.2. *In vivo*

All animal procedures were approved by the Yale University Institutional Animal Care and Use Committee. Glioma-bearing rats were generated by intracerebral injection of RG2 cells (10,000 cells) in anaesthetized (isoflurane, 2–3%) Fischer 344 rats (Charles River, CT, USA) as described previously (De Feyter et al., 2016). In short, using aseptic techniques, through a burr hole made in the skull cells suspended in 5 μL sterile saline were injected at a rate of 1 μL/min using a using a Hamilton syringe (26 gauge) attached to a motorized injector (World Precision Instruments, Sarasota, FL, USA), guided by a stereotaxic instrument (David Kopf Instruments, Tujunga, CA, USA). Peri-and post-operative care included the use of analgesics (Lidocaine: <7 mg/kg, Meloxicam: 1–2 mg/kg, and Buprenorphine: 0.01–0.05 mg/kg). After the tumor cell implantation animals were monitored daily for recovery.

For *in vivo* imaging, animals were anesthetized with isoflurane using ∼60% O_2_ and ∼40% N_2_O as carrier gas mixture, delivered through a nosecone. A heating pad was used to maintain body temperature at ∼37°C, and breathing rate was monitored continuously using a small pneumatic pillow sensor (SA Instruments Inc., Stony Brook, NY, USA). For intravenous (IV) infusion of Cho, catheters consisting of a 1 inch needle (30 gauge) and polyethylene tubing (PE10, Instech Laboratories, Plymouth Meeting, PA, USA) were placed in the rat lateral tail vein for imaging studies. For bench studies that included arterial blood sampling, both femoral artery and vein were canulated with P50 and P10 tubing, respectively.

**FIGURE 1:**
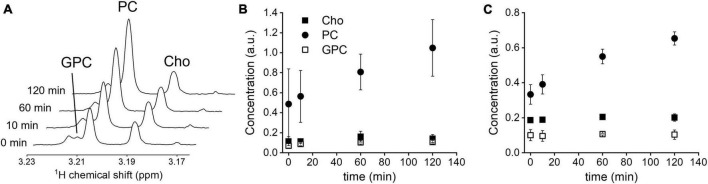
*In vitro* Cho uptake. **(A)** ^1^H NMR spectra of GL261 cell metabolite extracts at 0, 10, 60, and 120 min, zoomed in on the spectral region of the methyl group of choline-containing molecules. **(B)** Intracellular levels, normalized to creatine concentration, in RG2 cells (*n* = 3), and **(C)** GL261 cells (*n* = 3), during 120 min incubation with 1 mM unlabeled Cho. Cho, choline; PC, phosphocholine; GPC, glycerophosphocholine. Error bars = SD.

Unlabeled choline chloride (MilliporeSigma, Burlington, MA, USA), ^2^H_9_-choline chloride (Cambridge Isotopes Laboratories, Cambridge, MA, USA), or [1,1′,2,2′-^2^H_4_]-choline chloride (CDN Isotopes, Montreal, QC, Canada) was dissolved in sterile water (400 mM) and administered using a three-step bolus-continuous infusion protocol over 36 min (Figure 1). This infusion protocol resulted in a volume infused of 1.57 ml for a 250 gram rat, and a dose of 376 mg/kg body weight. Bench infusions with [1,1′,2,2′-^2^H_4_]-choline chloride were followed by euthanasia by focused beam microwave fixation of brain, either immediately after the infusion, or 20–24 h later (De Graaf et al., 2009). The use of focused beam microwave fixation results in loss of consciousness in <100 ms, and death in <1 s, and is an effective method to fix brain tissue *in vivo* for subsequent assay of enzymatically labile chemicals. Tumor and normal brain tissue were harvested, frozen in liquid N2 and stored at −80°C until further processing. Tissue metabolite samples were homogenized using a bead mill (Omni International, Kennesaw, GA, USA) and a standard methanol HCL extraction protocol (McNair et al., 2022). Samples were resuspended for ^2^H NMR in dedicated phosphate-buffered solution of deuterium-depleted water (Sigma-Aldrich, Burlington, MA, USA), containing deuterated formate (Sigma-Aldrich) and imidazole (Cambridge Isotopes Laboratories, Cambridge, MA, USA). Plasma samples were mixed with methanol for protein precipitation (Gowda and Raftery, 2014) dried under nitrogen flow, and prepared for NMR as described above for cell metabolite extracts.

Imaging studies were performed on an 11.74 T magnet interfaced to a Bruker Avance III HD spectrometer running on ParaVision 6 (Bruker Instruments, Billerica, MA, USA), as previously described (De Feyter et al., 2018). DMI acquisition in rats was performed with a 20 mm × 15 mm elliptical ^2^H surface coil, combined with two orthogonal 20 mm ^1^H surface coils driven in quadrature, used for anatomical imaging and shimming. After gradient-echo scout images were acquired to confirm positioning, contrast-enhanced T_1_-weighted (CE T1W) MRIs were acquired using a multi-slice spin-echo pulse sequence with a repetition time (TR) of 1,000 ms, echo time of 6.4 ms, 10–20 min after IV or subcutaneous injection of 150–200 μL of the T_1_ contrast agent gadopentetate dimeglumine (Magnevist^®^, Bayer, NJ, USA). Following anatomical MRIs 3D B_0_ mapping was performed (second order spherical harmonic shimming), resulting in circa 25–35 Hz water FWHM linewidth across a 7 × 5 × 10 mm = 750 μL volume. ^2^H MR signal acquisition was achieved with a pulse-acquire sequence extended with 3D phase-encoding gradients for spherical encoding during the initial 0.6 ms following excitation. DMI was acquired (TR = 400 ms, eight averages) at a 2.5 × 2.5 × 2.5 mm = 15.6 μL nominal spatial resolution as a 11 × 11 × 11 matrix in a 27.5 mm × 27.5 mm × 27.5 mm field of view using spherical k-space sampling, resulting in 36 min total scan time.

### 2.3. High resolution NMR

High resolution ^1^H and ^2^H NMR scans of cell and tissue metabolite extracts were performed on a 500–MHz Bruker Avance MR spectrometer (Bruker Instruments, Billerica, MA, USA) using a 5–mm probe optimized for ^1^H NMR, or a probe optimized for broadband acquisition for ^2^H NMR experiments. Proton NMR experiments were performed at 500.13 MHz, with a standard pulse-acquire sequence with pre-saturation water suppression (TR: 15 s, ns: 32). Deuterium NMR experiments were performed at 76.77 MHz, using a standard pulse-acquire sequence (TR: 1 s, ns: 7,200). Between 6 and 16 datasets were summed together.

### 2.4. Data processing

Deuterium metabolic imaging and high-resolution NMR data were processed using the in-house developed DMI Wizard and NMRWizard,^1^ respectively, graphical user interfaces in MATLAB (MathWorks, Natick, MA, USA). Peaks of the ^2^H MRSI spectra (DMI data) were quantified by spectral fitting through linear combination of two model spectra of the natural abundance water peak and the tCho peak. Spectral fitting included a variable zero order and fixed first order phase estimation. Conversion of signal amplitude to concentration was performed using the water signal as internal concentration standard of 12.5 mM, assuming brain and brain tumor water content of 80%, and 0.0156% deuterium natural abundance (Hagemann et al., 1970). No corrections for T1 relaxation were preformed given the comparable T1 values of water and tCho (Eng et al., 1990; De Feyter et al., 2018). Average values of brain tumor ^2^H-tCho were determined in voxels that visually contained at least ∼50% of tumor tissue, as detected on the 2D ^2^H MRSI grid that was overlaid on CE T1W MRI. Average values for normal brain were determined by selecting four voxels for each animal in the contralateral hemisphere on the 2D ^2^H MRSI grid that was overlaid on CE T1W MRI. For visualization, 2D interpolation was used by convolving the nominal DMI data with a Gaussian kernel, whereby the convolution provided an inherent Gaussian smoothing of 1.2–1.8–pixel widths. The interpolated DMI maps were overlaid on anatomical MRI as amplitude color maps (De Feyter et al., 2018). Cho, PC, and GPC levels detected with ^1^H or ^2^H high resolution NMR in cell, tissue metabolite extracts and plasma were quantified by peak integration after first or second order baseline correction. Cell extract metabolite levels were normalized to intracellular creatine levels and reported as arbitrary units. As a starting point the ^2^H NMR chemical shifts of ^2^H-labeled Cho metabolites were assumed to be similar to the ^1^H NMR chemical shifts. This assumption was validated by ^1^H and ^2^H NMR experiments of known individual solutions of Cho, PC, GPC, and [1,1′,2,2′-^2^H_4_]-Cho. The results of these experiments are described in the Supplementary material.

### 2.5. Statistics

All statistical tests were performed using SPSS (IBM, Armonk, NY, USA). Comparison of the ^2^H_9_-tCho concentration between tumor and normal brain, or between tumor tissue imaged during infusion vs. 20–24 h later, was performed using two-tailed, independent samples *T*-test. When data were first averaged per animal for display purposes *T*-test (Figure 5A) or Wilcoxon Rank test (Figure 5B) was used, as indicated.

## 3. Results

### 3.1. *In vitro*

Incubation of RG2 and GL261 cells with 1 mM of unlabeled Cho resulted in increased levels of intracellular PC, as detected with ^1^H NMR in cell pellet metabolite extracts (Figure 1). Introducing relatively high levels of Cho in the cell culture medium resulted in a steady rise of intracellular PC levels, with a near-linear increase during the 120 min incubation period, while Cho and glycerophosphocholine levels remained stable at the baseline level. These data agree with rapid phosphorylation of free Cho after transport into the cell, and Cho transport being the rate limiting step under these conditions (Katz-Brull and Degani, 1996).

### 3.2. *In vivo* DMI during infusion of ^2^H_9_-Cho

**FIGURE 2:**
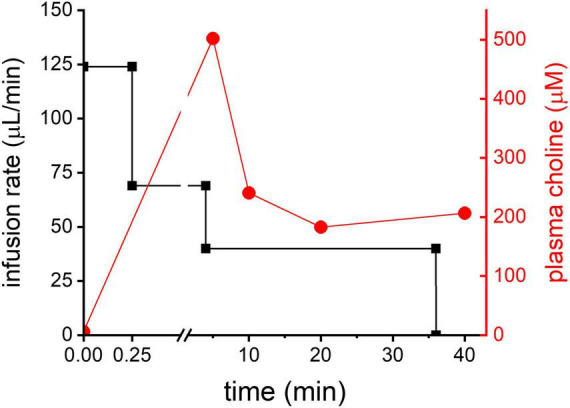
Cho infusion. In black: 3-step infusion protocol graphically shown for an animal with body weight of 250 g (left Y-axis). In red: plasma Cho concentration (mM) measured in one animal using the infusion protocol shown in black.

To test the feasibility of DMI of ^2^H_9_-Cho to generate tumor-to-brain image contrast the ^2^H labeled Cho was infused using a 3-step protocol for 36 min during the DMI data acquisition. The infusion protocol was based on an existing bolus-variable rate protocol for IV administration of ^13^C-labeled acetate, as used previously in our lab (Patel et al., 2010). The goal was to achieve a relatively quick increase followed by a steady level of plasma Cho for the duration of the DMI scan (=36 min). The protocol resulted in a moderate peak of plasma Cho 5 min after the start of the infusion followed by a leveling off after 10 min, as illustrated in Figure 2.

**FIGURE 3:**
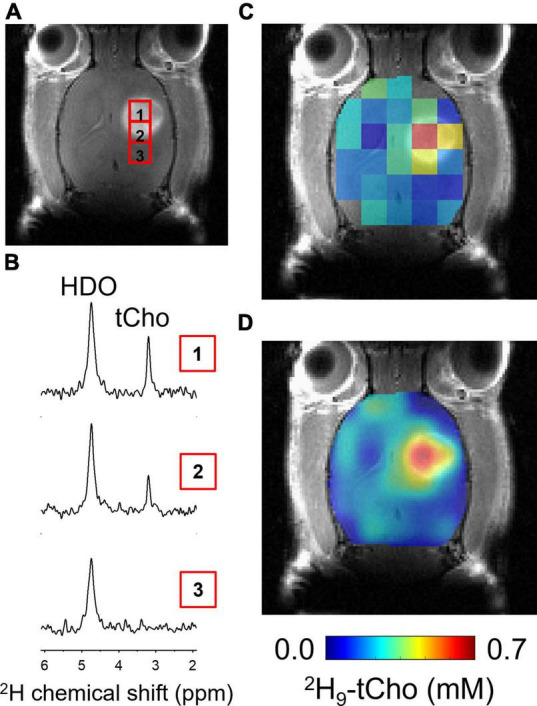
*In vivo* DMI during infusion of ^2^H_9_-Cho. **(A)** Coronal slice of CE T1W MRI from brain of RG2-bearing rat, showing the tumor lesion and individual voxel positions selected from the ^2^H MRSI grid. **(B)** Individual ^2^H spectra from voxel positions indicated on MRI shown in panel **(A)**. **(C)** Color-coded ^2^H MRSI grid overlaid on anatomical MRI. **(D)** Interpolated color-coded map based on data shown in panel **(C)**. Color scale applies to both C and F. tCho, total choline; HDO, natural abundant ^2^H-labeled H_2_O.

*In vivo* imaging studies included basic anatomical MRI in support of the metabolic imaging of deuterated Cho. All RG2-bearing rats showed a clear lesion on CE T1W MRI, confirming the compromised blood brain-barrier that is characteristic for GBM (Figure 3A). Figure 3 further illustrates the DMI acquisition in rat brain during the 36 min IV infusion of ^2^H_9_-Cho, and the data processing sequence. Individual ^2^H MR spectra are highlighted, selected from the indicated voxels within and immediately adjacent to the tumor lesion, and show the high level of ^2^H-tCho within the tumor and the lack thereof in the nearby normal-appearing brain. The high uptake and/or metabolism of deuterated Cho results in high contrast with normal brain, as visualized by the fully-processed DMI-based metabolic maps.

Quantified levels of ^2^H_9_-tCho concentration in voxels overlapping at least ∼50% of tumor lesion (*n* = 25), collected in eight different animals, was 0.47 ± 0.16 mM (mean ± SD), while in normal brain of the contralateral hemisphere (*n* = 32 voxels) the deuterated tCho concentration was 0.12 ± 0.06 mM (*T*-test, *p* < 0.0001). These data represent an average tumor-to-brain image contrast of 3.9 ± 1.4, expressed as the ratio of the average tCho concentration of tumor over normal brain.

### 3.3. *In vivo* DMI 24 h after infusion of ^2^H_9_-Cho

**FIGURE 4:**
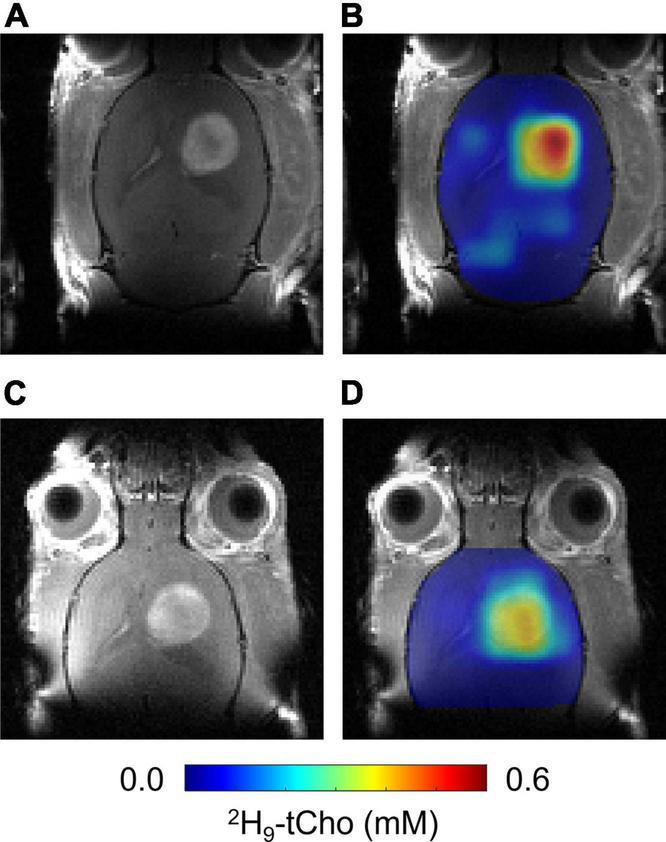
*In vivo* DMI 24 h after infusion of ^2^H_9_-Cho. **(A,C)** CE T1W MRI of same RG2-bearing rat acquired before 36 min infusion of ^2^H_9_-Cho **(A)** and 24 h later **(C)**. **(B,D)** Interpolated color-coded maps of ^2^H-tCho based on DMI data acquired during **(B)**, and 24 h after **(D)** infusion of ^2^H_9_-Cho, in the same animal.

**FIGURE 5:**
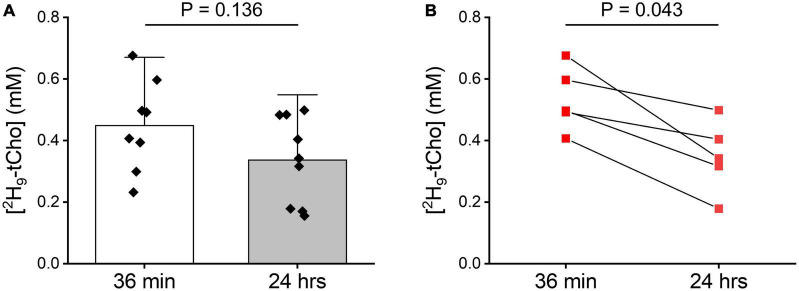
Tumor ^2^H-tCho concentration measured *in vivo*. **(A)** Average tumor ^2^H-tCho levels (mM) from DMI data acquired during the 36 min infusion of ^2^H_9_-Cho, and 20–24 h later. Bar graphs indicate mean and standard deviation, with individual data points overlaid. 36 min: *n* = 8; 24 h: (*n* = 9). Statistics based on *T*-test. **(B)** Subset of data shown in panel **(A)** of animals (*n* = 5) for which a pair of 36 in and 24 h data were collected. Statistics based on Wilcoxon Rank test.

The *in vitro* experiments were in agreement with fast phosphorylation of free Cho to PC, and with reported low values (100–180 μM) of Km of choline kinase α (Hudson et al., 2013). We anticipated that should subsequent metabolic conversions be relatively slow compared to Cho phosphorylation, then of ^2^H-labeled PC could be observed as ^2^H-tCho several hours after finishing the ^2^H_9_-Cho infusion. To test this concept DMI data were acquired during the described infusion protocol, and 20–24 h after the infusion. Figure 4 shows MRI and DMI data of the same animal acquired during and 24 h after the ^2^H_9_-Cho infusion. This example shows that even 1 day after the initial administration of deuterated Cho significant signal remains in the tumor lesion conferring good image contrast with normal brain. The concentration of ^2^H-tCho from DMI data acquired 20–24 h after infusion was 0.34 6± 0.16 mM (*n* = 36 voxels, *n* = 9 animals) in tumor, and 0.07 ± 0.04 mM (*n* = 36 voxels, *n* = 9 animals; *T*-test, *p* < 0.0001) in normal brain, resulting in a tumor-to-brain image contrast of 5.0 ± 2.3. The average ^2^H_9_-tCho concentration for tumor detected in each animal at 36 min and 24 h is summarized in Figure 5A which shows a 25% reduction in ^2^H_9_-tCho concentration over this period. The paired data (36 min and 24 h) from five animals are shown in Figure 5B.

### 3.4. ^2^H NMR after infusion of [1,1′,2,2′-^2^H_4_]-choline

**FIGURE 6:**
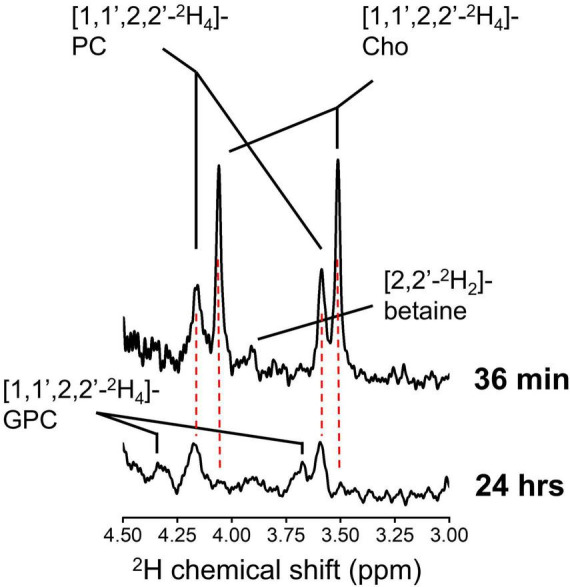
High resolution ^2^H NMR. ^2^H NMR spectra from tumor tissue metabolite extract collected from an animal euthanized immediately after 36 min infusion of ^2^H_9_-Cho **(top)**, and from an animal euthanized 24 h after the infusion **(bottom)**. Cho, choline; PC, phosphocholine; GPC, glycerophosphocholine.

The difference in chemical shifts of the ^2^H_9_-Cho, –PC, and –GPC trimethyl group around 3.2 ppm is less than 0.04 ppm, resulting in indiscernible overlapping peaks, both *in vivo* and in ^2^H high resolution NMR spectra. ^2^H NMR of ^2^H_9_-Cho is therefore unable to establish the contribution of different Cho metabolites to the ^2^H-tCho peak observed *in vivo*. This ambiguity can be resolved by infusion of [1,1′,2,2′,-^2^H_4_]-choline, as the ^2^H chemical shift differences between Cho, PC, and GPC on the C1 and C2 carbons is sufficient to allow clear separation. This approach is not viable for *in vivo* detection because the severalfold (9/2 = 4.5) reduced sensitivity due to the smaller number of deuterons per resonance. Figure 6 shows ^2^H NMR spectra acquired in metabolite extracts from RG2 tumor tissue collected immediately after, and 24 h after an infusion of [1,1′,2,2′-^2^H_4_]-choline. In tissue harvested at 36 min, the now resolved peaks can be assigned to free Cho and PC, and possibly a small contribution from labeled betaine. At 24 h, no free Cho is detected but peaks from the ^2^H-labeled PC and GPC can be observed. At 36 min the metabolite contribution to the total signal was 0.61 ± 0.02 for Cho/(Cho + PC) and 0.39 ± 0.02 for PC/(Cho + PC) (*n* = 3). For the samples collected after 24 h, the metabolite contribution to the total signal was 0.60 ± 0.00 for PC/(PC + GPC), and 0.40 ± 0.00 for GPC/(PC + GPC) (*n* = 2). Because the ^2^H-labeling patterns have no effect on uptake or metabolism of Cho the ratios of the different metabolites as fraction of the total signal can be extrapolated to the *in vivo* results using ^2^H_9_-Cho. Therefore, these results indicate that the DMI maps of tumor acquired during ^2^H_9_-Cho infusion represent ∼60% free Cho, ∼40% PC, and no GPC, whereas 1 day after infusion the tumor lesion is devoid of free Cho and contains a 60/40% contribution of the metabolites PC and GPC.

## 4. Discussion

In a preclinical rat model of GBM we explored the characteristics of imaging *in vivo* ^2^H-labeled Cho using DMI. The *in vivo* experiments were supported by cell culture studies, and high resolution ^2^H NMR in tissue metabolite extracts to better understand the implications of the *in vivo* DMI data acquired during or after infusion of ^2^H_9_-Cho. The primary outcome of the studies is the high tumor-to-brain image contrast provided by imaging ^2^H_9_-tCho with DMI. Further, the results indicate that by modifying the timing of the DMI data acquisition relative to the start of the ^2^H_9_-Cho infusion the DMI-based metabolic maps can be more representative of Cho transport or Cho metabolism.

The *in vitro* experiments in rat RG2 and mouse GL261 cells were performed with a high level of extracellular unlabeled Cho that resulted in a steady increase in intracellular PC during the 2 h of incubation, while intracellular Cho and GPC remained stable. The results imply avid Cho uptake by both GBM cell lines, and high capacity to phosphorylate the intracellular Cho to PC. The increase in PC concentration is somewhat unusual because most metabolic pathways can increase or decrease in flux rate without changing the level of the metabolite involved. While not directly comparable, the results and interpretation are in line with reports of cell studies using lower concentrations of extracellular Cho. These studies used ^2^H- or ^13^C-labeled Cho to study in much more detail its metabolism in breast cancer cell lines (Katz-Brull and Degani, 1996; Katz-Brull et al., 1998; Glunde et al., 2004). A direct comparison between the breast cancer cell studies and the present study in GBM cells is not possible but overall our findings are consistent with the notion that the phosphorylation rate of Cho by choline kinase α is much faster than the transport rate of Cho, leading to a fast conversion of Cho to PC, and the a net production and increase in PC pool size that we observe (Katz-Brull et al., 1998).

The administration of ^2^H_9_-Cho for the *in vivo* DMI experiments was done via intravenous infusion using a 3-step continuous infusion protocol. Any abnormal increase in plasma Cho concentration is counteracted by avid clearance by the kidneys. This leads to very high concentrations of Cho (>5 mM) in the kidneys which was previously shown with ^2^H MRS and ^2^H MRI (Eng et al., 1990). To compensate for the high clearance rate by the kidneys a continuous infusion was chosen instead of a single bolus to achieve an increased level of labeled plasma Cho for the 36 min DMI acquisition. This duration of the infusion protocol also keeps the plasma level of ^2^H-betaine, an oxidation product of Cho, low. Longer infusions typically result in a steady increase of plasma betaine, which can reach severalfold higher levels than Cho during a 2-h infusion (data not shown). The ^2^H-labeled trimethyl group from betaine has a chemical shift of 3.25 ppm which is close to Cho and PC peaks and would contribute to the ^2^H_9_-tCho signal detected *in vivo* with DMI. Lastly, a sudden increase in plasma Cho can result in a rapid and significant drop in blood pressure, and is therefore another reason to forego a high bolus infusion to administer ^2^H_9_-Cho for DMI studies (Savci et al., 2003).

Body water was used as an internal concentration standard, assuming a naturally abundant ^2^H fractional enrichment of 0.0156% (Hagemann et al., 1970). The latter value was reported as the average of samples from different oceans on earth, while the value determined in a sample representing Antarctic precipitation was 0.0089% (Hagemann et al., 1970). Yet another source lists the ^2^H fractional enrichment of “tank water” at 0.0112% (Rosman and Taylor, 1998). Assuming that body water reflects mostly tap (fresh) water intake by the animals, it is possible that the ^2^H_9_-tCho concentration is slightly overestimated. In studies where a highly accurate determination of ^2^H concentration of a metabolite is warranted, and where body water is used as internal concentration standard, a baseline estimation of the body water ^2^H fractional enrichment from a blood or urine sample would benefit the quantification.

Both the concurrent and delayed DMI acquisition resulted in metabolic maps with high contrast between tumor and normal brain, with the late DMI scan showing a ∼30% drop in ^2^H_9_-tCho concentration. Combined with the results of the ^2^H NMR on tumor tissue extracts we conclude that DMI during infusion of ^2^H_9_-Cho is weighted toward Cho uptake, with the ^2^H-tCho signal constituting 60% Cho and 40% PC, as measured immediately after 36 min of infusion. The delayed DMI acquisition results in maps weighted toward Cho metabolism with the ^2^H_9_-tCho consisting of 60% PC and 40% GPC. Given the patterns of ^2^H-labeling in Cho and its metabolites, and knowledge of the pathways involved in Cho metabolism, the delay between infusion and DMI acquisition could be better chosen than the 1 day wait period used in the present study. The fast rate of Cho phosphorylation and the short half-life of plasma Cho (15 min) (Buchman et al., 1994) suggest that DMI data acquired only a few hours after ^2^H_9_-Cho infusion would almost exclusively show labeled PC in the tumor lesion, and could thus be used to image choline kinase α activity. Further experiments are needed to optimize the scan timing in function of the Cho metabolite of interest and SNR of the delayed DMI acquisition. This approach would require tissue metabolite extracts to be analyzed with high resolution NMR, as presented in this study. Here we choose to use ^2^H NMR as this nucleus is the key to the *in vivo* imaging technique. However, experiments using ^13^C-labeled instead of deuterated Cho would be just as informative for the interpretation of DMI results, and could benefit from indirect detection with ^1^H-[^13^C] NMR which provides much higher sensitivity than ^2^H NMR.

The emphasis of this study is on the metabolism-based image contrast between normal brain and tumor, but the interpretation and understanding of the results builds on many years of research in Cho metabolism of cancer. This includes the early description of the “Kennedy pathway” of phospholipid synthesis that covers the anabolic enzymatic reactions from Cho to phosphatidylcholine, as well as the subsequent catabolic reactions that lead to the generation of GPC (Kresge et al., 2005; Glunde et al., 2011; Sonkar et al., 2019). This knowledge helps understand why the ^2^H-labeled GPC is observed 24 h but not after 36 min of ^2^H_9_-Cho infusion, because it takes a significant amount of time for the ^2^H_9_-Cho to be built in phosphatidylcholine before the breakdown of the latter results in labeled GPC, after a near complete turnover of the Kennedy pathway. Much of the Cho metabolism in cancer has been investigated using *in vivo* and *in vitro* NMR, both for detecting pool sizes as well as in combination with stable isotopes to provide detail on the actual metabolic fluxes. While the tCho pool size detection in patients with a brain tumor is one of the earliest applications of ^1^H MRS in patients (Bruhn et al., 1989), arguably more in-depth work has been performed by different groups on Cho metabolism in breast cancer (Katz-Brull and Degani, 1996; Katz-Brull et al., 1998, 2001, 2002b; Glunde et al., 2004, 2015; Cao et al., 2016; Cheng et al., 2017; Sonkar et al., 2019). Since *in vivo* experiments are by nature whole body experiments several aspects of the breast cancer experiments can be compared to the present work in brain tumor models. For example, in the work by Katz-Brull et al. (2001), where [1,2-^13^C]-choline was infused in a mouse model of breast cancer, limited uptake of Cho in normal brain is reported. This is in accordance with the very low background signal in the tCho-DMI maps, which contributes to high tumor-to-brain image contrast. ^13^C-labeled PC was the Cho metabolite in the breast tumor model with the highest level of labeling. While the timing of tumor tissue harvesting was different (2 h) than in the present study, at least qualitatively these observations agree with the observation and interpretation of fast synthesis of PC from the infused labeled Cho (Katz-Brull et al., 2001). Other work focused on the potential of targeting Cho metabolism as therapy and as biomarker of response (Cheng et al., 2017). Here the observation was made that detecting the tCho pool size alone can obscure opposite changes in PC and GPC following treatment in breast cancer, and that ideally PC and GPC would be detected individually (Cheng et al., 2017). If tCho-DMI is shown to be applicable in breast cancer, the timing of a tCho-DMI scan could be optimized such that mostly or exclusively ^2^H-labeled PC is detected, and possibly function as an improved readout of therapy effect.

DMI acquisition has already been used in human subjects by different research groups and at different magnetic field strengths, and is thus proven to be translatable (Ruhm et al., 2021; Kaggie et al., 2022; Roig et al., 2022). Examples of DMI combined with oral intake of deuterated glucose in patients with brain tumors has also shown high tumor-to-brain image contrast (De Feyter et al., 2018). Because normal brain also takes up high levels of glucose here the image contrast depends on the difference in how the labeled glucose is metabolized in tumor and normal brain. In the case of ^2^H_9_-Cho, the contrast between normal brain and brain tumor is already at the level of uptake of the substrate, and therefore DMI of ^2^H_9_-Cho could potentially provide higher image contrast than ^2^H-glucose. Moreover, both deuterated glucose and Cho could be combined in a single study. Veltien et al. (2021) have shown in a mouse model of renal cell carcinoma that ^2^H_9_-Cho and [6,6′-^2^H_2_]-glucose can be co-infused and thus in a single DMI scan provide information of two separate, cancer-specific metabolic pathways.

Successful translation of imaging Cho uptake and/or metabolism with DMI also depends on administration of the ^2^H-labeled Cho. Intravenous infusion is a possibility and used in the clinic, for example in patients receiving parenteral nutrition, yet at doses that are severalfold lower than used in the present animal study (Buchman et al., 1994, 1995). Potential toxic side effects of high Cho administration are the consequence of Cho mimicking acetylcholine, the primary neurotransmitter of the parasympathetic nervous system. Effects of too high of an acute Cho dose include a rapid drop in blood pressure and stimulation of skeletal muscle that can result in twitching (Lott and Jones, 2022). These effects can be countered by pre-emptive administration of a cholinergic antagonist such as atropine, as used in previous imaging studies that relied on the infusion of Cho (Katz-Brull et al., 2001; Veltien et al., 2021). In the present study we did not observe obvious signs of Cho toxicity without the use of atropine, possibly because the relatively slow infusion rate compared to bolus injections of Cho chloride solutions. Atropine also can cross the blood-brain barrier and thus can counteract any cholinergic effects in the brain, and even brain tumor. Cho is not only the precursor for acetylcholine but also an agonist of the alpha 7 and alpha 9-nicotinic receptor (Albuquerque et al., 1997). *In vitro* studies using GBM cell lines suggest a proliferative effect of Cho, not by supporting phospholipid synthesis but by binding to α7- or α9-containing receptors (Pucci et al., 2021). Recent studies have also suggested a possible function for acetylcholine in modulating invasion by GBM cells through extracellular matrices, and indicated the presence of acetylcholine receptors in human GBM based on RNA sequencing data available in human genome atlases (Thompson and Sontheimer, 2019). Because tCho-DMI-based metabolic maps are unlikely to be affected by atropine it is recommended to include atropine in an intravenous infusion protocol of ^2^H_9_-choline chloride to counter cholinergic stimulation to avoid both systemic effects as well as possible tumor-specific consequences.

Safety concerns about Cho are less worrisome for oral administration, for which upper level dose recommendations are 3.5 g per day for adults, and anecdotal evidence exists of higher doses being used (Choline, 2014; Office of Dietary Supplements, 2022). Further development of tCho-DMI should incorporate testing and optimization of oral Cho loading regimes, including the use of multiple oral doses over several days. The present data indicate that the ^2^H label is to some extent “trapped” in PC and GPC in the 24 h following administration, and since the *in vitro* data suggest that the PC pool can be increased, a high level of labeled PC and GPC could possibly be achieved with serial low oral doses of ^2^H_9_-Cho. Each oral dose would lead to a short increase in plasma ^2^H_9_-Cho, leading to subsequent transport into the tumor, conversion to ^2^H_9_-PC that could be detected with DMI. Based on the present data the washout of ^2^H_9_-tCho from the tumor is anticipated to be much slower than disappearance of plasma Cho, and thus each subsequent oral dose would add to the existing level of ^2^H-labeling in the tumor and increase the accumulated ^2^H-tCho signal to be observed with DMI.

A limitation of this work is the use of only a single animal model of GBM. Other rodent models of high-grade tumors should be investigated, as well as lower grade brain tumor models with intact blood-brain barrier to characterize its role in tumor Cho uptake. Additional preclinical studies are needed to test the value of DMI of ^2^H_9_-Cho uptake and/or metabolism as an imaging tool that can assist with evaluating treatment effect and distinguishing true progression from pseudoprogression, two main aspects of GBM management that are very difficult to assess with existing imaging modalities. More work is also needed to establish the specificity of high ^2^H_9_-tCho and evaluate to what extent other proliferating cell types, such as immune cells, could contribute to the observed metabolic image contrast. Lastly, other types of cancer than brain tumors could also show significant ^2^H_9_-tCho-based image contrast, which could be helpful in disease management.

This study showed the potential of imaging deuterated Cho with DMI in rodent GBM and illustrates how timing of the DMI scan can put emphasis on detecting ^2^H_9_-Cho uptake or ^2^H_9_-Cho metabolism. High image contrast between tumor lesions and normal brain was observed. The proven translatability of the DMI imaging technology, together with the safe use of deuterium, and Cho as nutritional supplement, emphasize the potential of this approach for implementation in future studies of patients.

## Data availability statement

The raw data supporting the conclusions of this article will be made available by the authors, without undue reservation.

## Ethics statement

This animal study was reviewed and approved by the Institutional Animal Care and Use Committee (IACUC) of Yale University.

## Author contributions

KI, MT, and HD designed and performed the experiments and analyzed the data. HD wrote and edited the manuscript. KB and RG edited the manuscript. RG developed methods for data acquisition and processing. All authors contributed to the article and approved the submitted version.

## Footnotes

1
https://medicine.yale.edu/lab/dmi/
