# Supplementary material for: Mapping of exogenous choline uptake and metabolism in rat glioblastoma using deuterium metabolic imaging (DMI)
Source: Front Cell Neurosci. 2023 Apr 28;17:1130816. doi: 10.3389/fncel.2023.1130816 (PMC10175635; doi:10.3389/fncel.2023.1130816)
Supplement: Supplementary file 1 [file Data_Sheet_1.PDF]

Mapping of exogenous choline uptake and metabolism in rat glioblastoma using deuterium metabolic imaging (DMI)

Kevan L. Ip[1], Monique A. Thomas[1], Kevin L. Behar[2], Robin A. de Graaf[1,3], Henk M. De Feyter[1*]

Supplemental Materials.

Chemical shifts of proton/deuterium atoms at similar positions in a molecule are generally considered to have near identical chemical shifts in ^{1}H and ^{2}H NMR spectra [1]. To confirm that the ^{1}H and ^{2}H NMR chemical shifts of the $-CH_2-O$ and $N-CH_2$ resonances of choline and its metabolites were close, samples were prepared in a buffer that contained unlabeled and deuterated sodium formate to function as a chemical shift reference on both ^{1}H and ^{2}H frequencies. Individual samples of unlabeled choline (Cho), phosphocholine (PC), glycerophosphocholine (GPC) and [1,1',2,2'-$^{2}H_4$]-choline ($^{2}H_4$-Cho) were prepared, to be compared with the existing samples of metabolite extracts from RG2 tumor tissue harvested 36 min and 24 hours after infusion of [1,1',2,2'-$^{2}H_4$]-choline. When formate was referenced at 8.444 ppm for both nuclei a misalignment of 0.06 ppm became apparent between the ^{1}H NMR spectrum of the unlabeled Cho and the ^{2}H NMR spectrum of $^{2}H_4$-Cho (Fig. S1). Using the unified scale for reporting chemical shift indicated that the chemical shift of ^{2}H-formate should be 8.436 ppm instead of 8.444 ppm as used in ^{1}H NMR [2]. Yet, this difference was opposite and smaller than the 0.06 ppm discrepancy initially observed, suggesting a 0.06 ppm chemical shift difference between ^{1}H and ^{2}H for the $-CH_2-O$ and $N-CH_2$ resonances. When this chemical shift difference was taken into account by using 8.444 ppm (^{1}H) and 8.505 ppm (^{2}H) for formate, the ^{1}H NMR spectra of the Cho, PC and GPC solutions aligned perfectly with the ^{2}H NMR spectra of the $^{2}H_4$-Cho solution and the tissue samples for the $-CH_2-O$ and $N-CH_2$ peaks. The small difference in chemical shift between the protonated and deuterated molecules is within the range described by Saunders et al. for a series of other molecules (Table 1 in [3]). The ^{1}H and ^{2}H NMR chemical shifts we observed for choline and its metabolites (using formate as reference at 8.444 ppm) were summarized in Table S1.

1. Mantsch, H. H., Saitô, H. & Smith, I. C. P. Deuterium magnetic resonance, applications in chemistry, physics and biology. *Prog. Nucl. Magn. Reson. Spectrosc.* **11**, 211–272 (1977).
2. Harris, R. K., Becker, E. D., Cabral de Menezes, S. M., Goodfellow, R. & Granger, P. NMR Nomenclature: Nuclear Spin Properties and Conventions for Chemical Shifts. IUPAC Recommendations 2001. *Solid State Nucl. Magn. Reson.* **22**, 458–483 (2002).
3. Saunders, M., Saunders, S. & Johnson, C. A. A new NMR method for measuring the difference between corresponding proton and deuterium chemical shifts. Isotope effects on exchange equilibria. *J. Am. Chem. Soc.* **106**, 3098–3101 (1984).

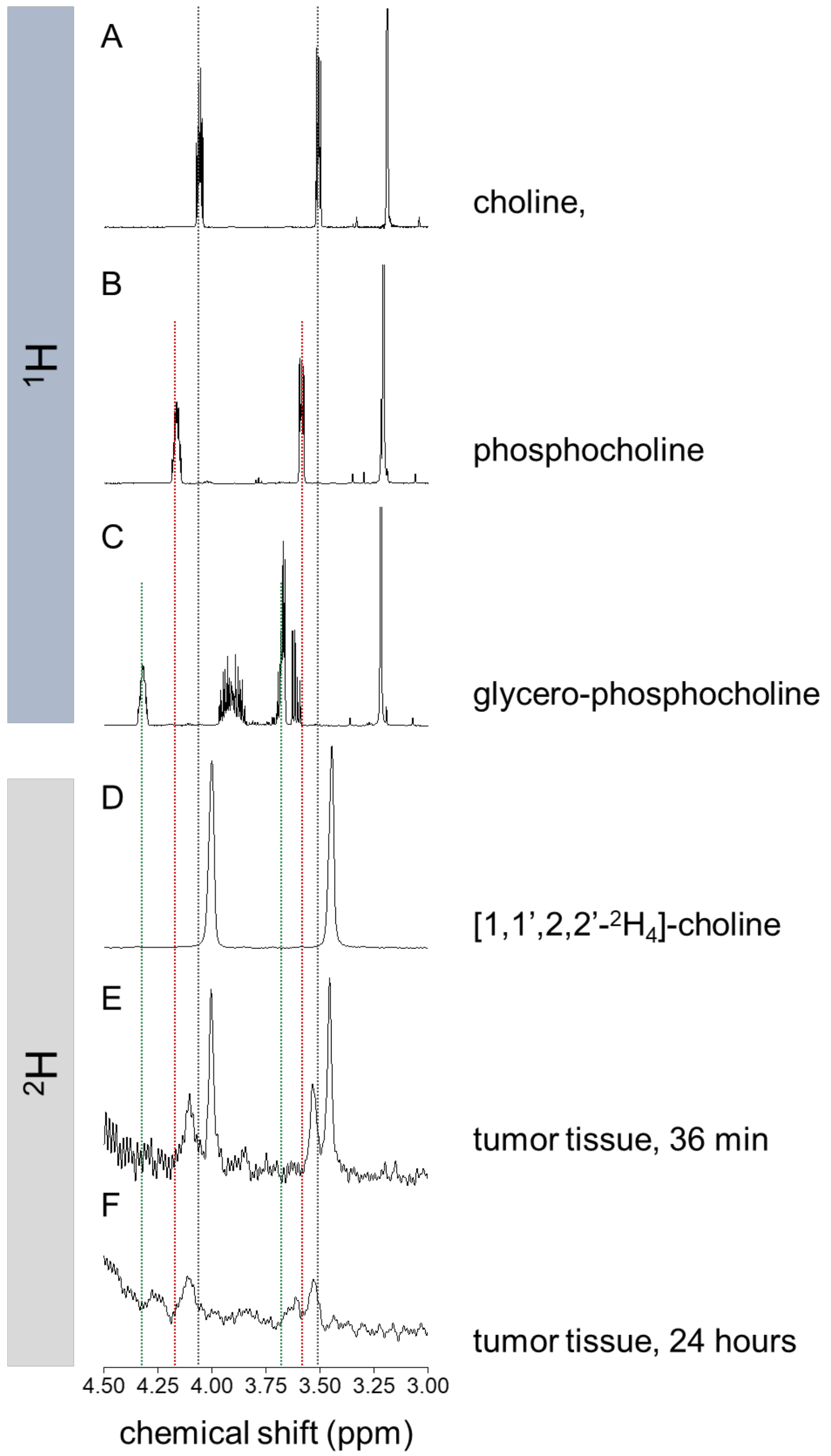

Figure S1. High resolution ^{1}H and ^{2}H NMR of choline + metabolites. A – C): ^{1}H NMR spectra of 100 mM choline chloride (A), phosphocholine (B) and glycerophosphocholine (C), at pH 7.2. D – F) ^{2}H NMR spectra acquired in solution of [1,1'2,2'-$^2H_4$]-choline chloride (D) and metabolite extract of RG2 tumor tissue samples harvested immediately after (E) or 24 hours (F) after 36 min of intravenous infusion of [1,1'2,2'-$^2H_4$]-choline chloride. All spectra used formate at a chemical shift of 8.444 ppm as reference. The dotted drop lines indicate the ~0.06 ppm difference in chemical shift between the ^{1}H and ^{2}H resonances of protonated and deuterated molecules.

Table S1

			Chemical shift (ppm)		
	nucleus	formate	$N(CH_3)3$	NCH_2	CH_2O
choline	^1H	8.444	3.19	3.51	4.06
phosphocholine	^1H	8.444	3.21	3.59	4.17
glycerophosphocholine	^1H	8.444	3.22	3.68	4.32
[1,1,2,2,-$^2H_4$]-choline	^2H	8.444	n.d.	3.46	4.01
[1,1,2,2,-$^2H_4$]-phosphocholine	^2H	8.444	n.d.	3.54	4.10
[1,1,2,2-$^2H_4$]-glycerophosphocholine	^2H	8.444	n.d.	3.62	4.26

n.d.: not determined.
